# Reciprocal Tripartite Interactions between the *Aedes aegypti* Midgut Microbiota, Innate Immune System and Dengue Virus Influences Vector Competence

**DOI:** 10.1371/journal.pntd.0001561

**Published:** 2012-03-06

**Authors:** Jose Luis Ramirez, Jayme Souza-Neto, Rolando Torres Cosme, Jose Rovira, Alma Ortiz, Juan M. Pascale, George Dimopoulos

**Affiliations:** 1 W. Harry Feinstone Department of Molecular Microbiology and Immunology, Bloomberg School of Public Health, Johns Hopkins University, Baltimore, Maryland, United States of America; 2 Gorgas Memorial Institute for Health Research, Panama City, Panama; Monash University, Australia

## Abstract

Dengue virus is one of the most important arboviral pathogens and the causative agent of dengue fever, dengue hemorrhagic fever, and dengue shock syndrome. It is transmitted between humans by the mosquitoes *Aedes aegypti* and *Aedes albopictus*, and at least 2.5 billion people are at daily risk of infection. During their lifecycle, mosquitoes are exposed to a variety of microbes, some of which are needed for their successful development into adulthood. However, recent studies have suggested that the adult mosquito's midgut microflora is critical in influencing the transmission of human pathogens. In this study we assessed the reciprocal interactions between the mosquito's midgut microbiota and dengue virus infection that are, to a large extent, mediated by the mosquito's innate immune system. We observed a marked decrease in susceptibility to dengue virus infection when mosquitoes harbored certain field-derived bacterial isolates in their midgut. Transcript abundance analysis of selected antimicrobial peptide genes suggested that the mosquito's microbiota elicits a basal immune activity that appears to act against dengue virus infection. Conversely, the elicitation of the mosquito immune response by dengue virus infection itself influences the microbial load of the mosquito midgut. In sum, we show that the mosquito's microbiota influences dengue virus infection of the mosquito, which in turn activates its antibacterial responses.

## Author Summary

Dengue virus is transmitted by *Aedes* mosquitoes. During their lifecycle, mosquitoes are exposed to a variety of microbes, and many of them inhabit the mosquito midgut, thereby sharing the same environment with ingested pathogens. The mosquito midgut is the site of multiple reciprocal interactions between the mosquito, its commensal bacteria, and ingested pathogens that will ultimately influence the level of pathogen infection and transmission. In this study the authors addressed the reciprocal interactions between the *Aedes* immune system, dengue virus and mosquito midgut microbiota using molecular and microbiological assays. The study showed that certain field-derived bacterial isolates of the mosquito midgut exert a detrimental effect on dengue virus infection. This effect is at least partly manifested through the action of the mosquito immune system which is activated by microbes. Conversely, dengue virus infection induces immune responses in the mosquito midgut tissue that act against the natural mosquito midgut microbiota. This study contributes to our understanding of dengue virus infection in *Aedes* mosquitoes, which may aid towards the development of novel biocontrol strategies to halt dengue transmission.

## Introduction

Dengue has become one of the most important arboviral diseases, with infections rising at an alarming rate [1]. The dengue virus is transmitted by two highly anthropophilic mosquitoes, *Aedes aegypti* and *Ae. albopictus*. Although advances have been made toward the development of a vaccine, no cure for dengue is currently available [1]. Current methods are aimed at lowering the vector population through insecticide use, but there are concerns about the environmental impact of this approach as well as the rapid development of resistance in mosquitoes [2]. These setbacks have underscored the need for the development of additional methods to control dengue transmission.

In the past decade, there has been a notable increase in research aiming at the potential application of microbes to control the transmission of vector-borne pathogens [3]. These studies have been encouraged by the fact that pathogens and microbes inhabit the same environment prior to infection (the arthropod midgut) and on the observation that pathogen infection is decreased in vectors harboring particular bacterial symbionts.

In fact, the midgut is the site of multi-taxon interactions that include the arthropod vector (host), vertebrate blood factors, the pathogen (virus or parasite), and other symbiotic microbes. Although there is growing interest in these associations, our understanding of how these interactions at the molecular level and how they affect vector physiology and influence vector competence is still very basic. It has been shown that some of these interactions involve insect immune factors such as lectins, antimicrobial peptides, digestive enzymes, nitric oxide, and the prophenoloxidase complex [4]–[6]. Other factors and mechanisms that have been suggested to contribute to these interactions and to modulate vector competence include: bacteria-derived cytolisins (hemolysins), siderophores, proteases, anti-parasitic factors, and secondary metabolites [4].

The purpose of the present study was to analyze the cultivable endogenous microbial flora of field mosquitoes collected from dengue-endemic areas in Panama and to assess their influence on the mosquito immune system and dengue virus infection. The incidence of dengue in Panama is the fifth-highest in Central America, and all four dengue virus serotypes are currently present in the country [7]. Molecular and infection assays have revealed intricate reciprocal interactions among the mosquito, the dengue virus, and its microbiota, with some bacterial isolates significantly affecting vector competence by reducing dengue virus infection of the midgut. In turn, the activation of the mosquito immune system by dengue virus infection alters the mosquito's immune homeostasis in the midgut, thereby affecting its microbiota.

## Methods

### Rearing and collection of field *Ae. aegypti* mosquitoes

The mosquito *Ae. aegypti* Rockefeller strain used in this study was maintained on a 10% sugar solution at 27°C and 95% humidity with a 12-h light/dark cycle according to standard procedures. Sterile cotton, filter paper, and sterilized nets were used to maintain maximum sterility of the cages.

The *Ae. aegypti* mosquitoes for this study were collected outdoors with BG-sentinel mosquito traps and indoors with mosquito aspirators from three regions: Panama Centro (Panama City, Felipillo), Panama Oeste (Chorrera), and Chiriquí (David). These sites were chosen on the basis of their prevalence of dengue fever and dengue hemorrhagic fever cases in the last 3 years and on mosquito surveys conducted by the Center for Mosquito Surveillance, Ministry of Health (MINSA, from its Spanish acronym). Peridomestic collection of mosquitoes in selected areas was conducted in the early hours of the morning (5:30 to 6:30am) and late afternoon (6:00 to 7:30pm). At least 10 mosquitoes per site were collected and processed.

### Isolation and characterization of mosquito midgut bacteria

The collected mosquitoes were transported back to the laboratory, chilled on ice, and identified at the species level using a stereoscope and the taxonomic keys of Galindo and Adames [8] and Rueda [9]. Following species confirmation, mosquitoes were surfaced-sterilized by dipping and shaking them in 75% ethanol for 2 min and rinsing them with 1× PBS twice for 1 min each. Midguts were then dissected from each individual mosquito over a sterile glass slide containing a drop of 1× PBS, then transferred to a microcentrifuge tube containing 150 µl of sterile PBS and macerated for 30 sec. Three 10-fold serial dilutions were then plated on LB agar and kept at room temperature for 48 h. Initial isolation was based on morphology, color, and size of colony (Figure S1), and then followed by molecular identification via 16s rRNA gene sequencing. The primers used to amplify the 16s rRNA gene were those reported by Cirimotich et al [10] : forward, AGAGTTTGATCCTGGCTCAG; and reverse (degenerate), TACGGYTACGCTTGTTACGACT. PCR conditions were used according to the Platinum Pfx DNA Polymerase (Invitrogen) protocol. PCR amplification was done with an initial denaturation of 2 minutes at 94°C, and 40 cycles with a denaturation step at 94°C for 30 seconds, an annealing step at 58°C for 30 seconds and an extension step at 72°C for 1 minute.

Bacterial 16s rRNA gene sequences were manually curated and assembled from forward and reverse primer-generated sequences. Curated sequences were then aligned and compared to available bacterial sequences in GenBank and in the Ribosomal Database Project (RDP Release 10, http://rdp.cme.msu.edu/). A bacterial phylogenetic tree was constructed using the Ribosomal Database Project “Tree Builder” program, which uses bootstrap sampling and the Weighbor weighted neighbor-joining tree-building algorithm to best estimate the phylogenetic position of a sequence.

### Mosquito antibiotic treatment and reintroduction of bacteria

Mosquitoes were rendered free of cultivable bacteria (designated as aseptic) by maintaining them on a 10% sucrose solution with 20 units of penicillin and 20 µg of streptomycin from the first day post-eclosion until 2 days prior to challenge. They were then maintained for 1 day on sterile water and starved for 24 h prior to dengue virus infection. Effectiveness of the antimicrobial treatment was confirmed by colony forming unit (CFU) assays prior to blood-feeding or bacterial challenge.

Two types of bacterial reintroduction were tested: via blood meal and via sugar meal. Reintroduction of bacteria through the blood meal was accomplished by first treating the mosquitoes with antibiotics and then providing them with cotton balls moistened with sterile water for 24 h post-antibiotic treatment. Mosquitoes were starved overnight and fed on a mixture containing 50% of a given bacterium suspended in 1× PBS (final concentration: OD_600_ = 1, for controls only 1× PBS was added), 25% of MEM (devoid of any antibiotics), 25% human commercial blood, and 10% human serum. Mosquitoes were cold-anesthetized, and the fully fed mosquitoes were separated and provided with a dengue virus-infectious blood meal 4 days after bacterial reintroduction. Infection phenotype assays were performed as previously reported [11] and as described below.

Following the bacterial reintroduction via blood meal, a subset of bacteria showing an effect on dengue virus infection was further tested through reintroduction via a sugar meal, which would more closely resemble natural bacterial acquisition. The bacteria were reintroduced through a sugar meal by first treating mosquitoes with antibiotics for the first 2–3 days after emergence and then providing them with a sterile 10% sugar meal for 24 h after antibiotic treatment. Mosquitoes were then starved overnight and fed on cotton strips moistened with a bacterial suspension diluted in 3% sucrose solution and suspended in a 1.5-ml microcentrifuge tube. *Proteus sp.* and *Pantoea sp.* were used at an OD_600_ of 1.00. Bacterial concentrations used to infect mosquitoes were determined on the basis of the average bacterial load for each bacterial strain found in the midgut of field-collected mosquitoes. Initial assessment of sugar meal acquisition and the location of the sugar meal following ingestion were made by providing a group of mosquitoes with a sugar solution dyed with blue food colorant. Midguts and crops of exposed mosquitoes were dissected at 6 and 24 h.

### Cell culture maintenance and DENV-2 infections

Dengue virus serotype 2 (New Guinea C strain, DENV-2) was propagated in the C6/36 cell line according to standard conditions [11]. In brief, 0.5 ml of virus stock was used to infect a 75-cm^2^ flask of C6/36 cells at 80% confluence. Infection was allowed to proceed for 5–7 days, at which time the cells were harvested with a cell scraper and lysed by freezing and thawing in dry CO_2_ and a 37°C water bath, centrifuged at 800 g for 10 min, and mixed 1∶1 with commercial human blood. The infectious blood meal was maintained at 37°C for 30 min prior to feeding 5- to 7-day-old mosquitoes.

### Mosquito dissections and dengue virus titration of infected midguts

Infected mosquitoes were collected at 7 days post-infection and surface-sterilized by dipping them in 70% ethanol for 1 min, then rinsing them twice in 1× PBS for 2 min each. Midgut dissection was performed in one drop of 1× PBS under sterile conditions, and the midgut was transferred to a microcentrifuge tube containing 150 µl of MEM. Midguts were homogenized using a Kontes pellet pestle motor and stored at −80°C until used for virus titration.

Dengue virus titration of infected midguts was done as previously reported [11], [12].The infected midgut homogenates were serially diluted and inoculated into C6/36 cells in 24-well plates. After an incubation of 5 days at 32°C and 5% CO_2_, the plates were fixed with 50%/50% methanol/acetone, and plaques were assayed by peroxidase immunostaining using mouse hyperimmune ascitic fluid specific for DENV-2 as the primary antibody and a goat anti-mouse HRP conjugate as the secondary antibody. Also, where indicated, dengue virus titration of infected midguts was conducted in BHK-21 cells. At 5 days post-infection, the 24-well plates were fixed and stained with crystal violet. Plaques (formed by cells with cytopathic effect, CPE) were counted and analyzed.

### Real-time qPCR assays

Real-time PCR assays were conducted by first treating the RNA samples with Turbo DNase (Ambion, Austin, Texas, United States); they were then reverse-transcribed using M-MLV reverse transcriptase (Promega, USA). The real-time PCR assays were performed using the SYBR Green PCR Master Mix kit (Applied Biosystems, Foster City, California, USA) in a 20-µl reaction volume, and all samples were tested in duplicate. The ribosomal protein S7 gene was used for normalization of the cDNA templates. The primer sequences used in these assays are listed in Table S1.

### RNAi-based gene-silencing assays

RNA interference assays (RNAi-based gene silencing) were conducted as previously reported [11]. In brief, 69 nl of dsRNA (3 ug/µl) re-suspended in water was injected into the thorax of cold-anesthetized 3- to 4-day-old female mosquitoes using a nano-injector. Three days after injection and gene-silencing validation, the mosquitoes were allowed to feed on a dengue virus-laden blood meal. Dissection of midguts and virus titration were carried out as described above. The primer sequences used are listed in Table S2.

### Statistical analysis

Real-time PCR assays were normalized and standardized according to Willems et al. [13]. Mann-Whitney U-tests and one-way ANOVA with Dunnett's post-test were used when appropriate. Statistical analyses were conducted using the GraphPad Prism statistical software package (Prism 5.05; GraphPad Software, Inc., San Diego, CA). Statistical significance is indicated with asterisks: *, p<0.05; **, p<0.01; ***, p<0.001.

## Results

### The cultivable mosquito midgut microbiome

**Figure 1:**
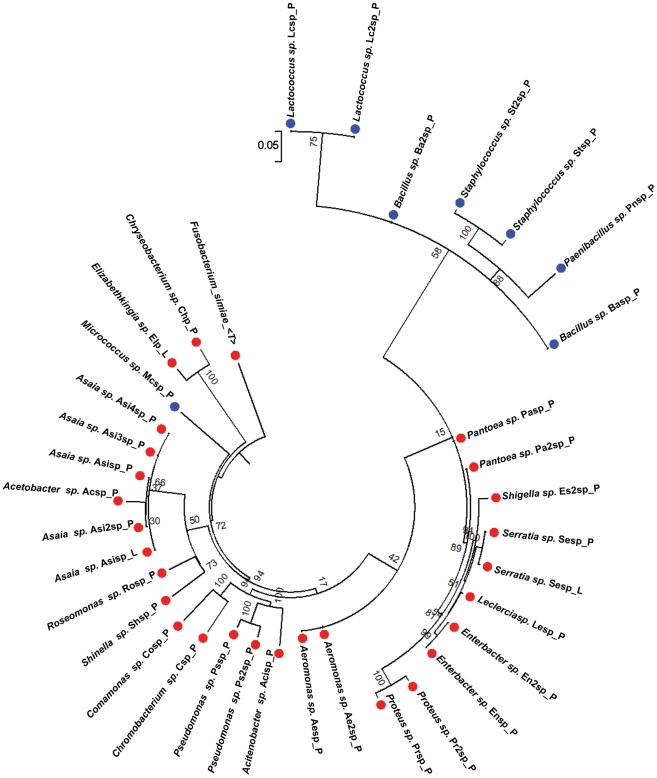
Phylogenetic tree of the field and laboratory-reared *Ae. aegypti* cultivable midgut microbiota. Red dots: Gram-negative, blue dots: Gram-positive. Phylogenetic tree constructed from the alignment of complete 16s rRNA sequences using the Weighbor weighted neighbor-joining algorithm from the Ribosomal Database Project, with *Fusobacterium simiae* as an out-group. The phylogenetic tree was generated using MEGA (v5).

**Figure 2:**
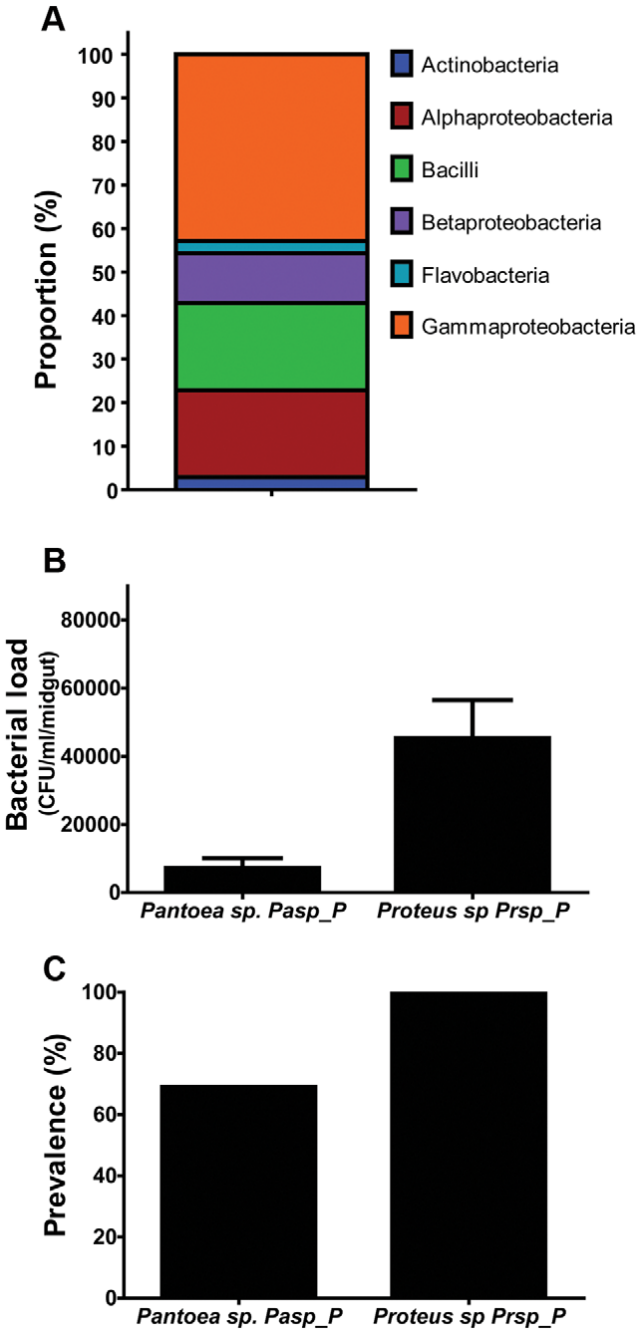
Characteristics of cultivable bacteria from the midgut of field-collected mosquitoes. (**A**). Proportions of bacterial phylogenetic classes in the mosquito midgut. (**B**). Bacterial load and (**C**) Bacterial prevalence in the mosquito midgut at 3 days post-bacterial acquisition via sugar meal. This coincides with the time when mosquitoes were exposed to an infectious blood meal.

To investigate the cultivable bacterial species composition of midguts from field-caught adult female *Ae. aegypti*, we conducted mosquito collections in dengue-endemic areas of Panama. The field-captured mosquitoes were surfaced-sterilized and dissected, and their midguts were homogenized and plated on rich culture medium. We isolated 40 distinct bacterial isolates on the basis of colony morphology and successfully characterized 34 of them. The bacteria isolated from the midguts of the field-collected mosquitoes were mostly Gram-negative, with no overrepresentation of a single genus (Table 1, Figure 1). Six bacterial genera have been previously isolated from mosquitoes, *Asaia spp*. [14], [15], *Aeromonas spp*., *Enterobacter spp*. [16], *Paenibacillus spp*. [16], *Proteus spp*. [17], and *Comamonas spp*. [18]. The isolated bacteria belonged to six phylogenetic classes, with the most dominant being the Gammaproteobacteria, the Betaproteobacteria, the Bacilli, and the Alphaproteobacteria (Figure 2A).

**Table 1:** Phylogenetic affiliations of cultivable bacterial isolates.

Bacterial Division (class)	Genus	Closest relative	Gram staining	% Identity	Source	Geographic Region
Actinobacteria	*Micrococcus sp.*	*Micrococcus sp. MOLA*	Gram (+)	99%	Field-mosquito	Panama Metro
Alphaproteobacteria	*Acetobacter sp.*	*Acetobacter ghanensis strain 430A*	Gram (−)	99%	Field-mosquito	Panama Metro
Alphaproteobacteria	*Asaia sp.*	*Asaia Krungthepensis isolate AE76*	Gram (−)	99%	Field-mosquito	Panama Metro
Alphaproteobacteria	*Asaia sp.*	*Asaia Krungthepensis*	Gram (−)	99%	Field-mosquito	Panama Metro
Alphaproteobacteria	*Asaia sp.*	*Asaia bogorensis*	Gram (−)	99%	Field-mosquito	Chorrera
Alphaproteobacteria	*Asaia sp.*	*Asaia bogorensis*	Gram (−)	99%	Lab-mosquito	Maryland, USA
Alphaproteobacteria	*Asaia sp.*	*Asaia bogorensis*	Gram (−)	100%	Field-mosquito	Chorrera
Alphaproteobacteria	*Roseomonas sp.*	*Roseomonas sp. QQDP511*	Gram (−)	98%	Field-mosquito	Panama Metro
Bacilli	*Bacillus sp.*	*Bacillus subtilis strain 22*	Gram (+)	99%	Field-mosquito	Panama Metro
Bacilli	*Bacillus sp.*	*Bacillus subtilis*	Gram (+)	99%	Field-mosquito	Chorrera
Bacilli	*Staphylococcus*	*Staphylococcus capprae*	Gram (+)	99%	Field-mosquito	David
Bacilli	*Staphylococcus*	*Staphylococcus capprae*	Gram (+)	99%	Field-mosquito	Panama Metro
Bacilli	*Lactococcus sp.*	*Lactococcus lactis CV56*	Gram (+)	99%	Field-mosquito	Chorrera
Bacilli	*Lactococcus sp.*	*Lactococcus lactis*	Gram (+)	99%	Field-mosquito	Chorrera
Bacilli	*Paenibacillus sp*	*Paenibacillus sp. GP26-03*	Gram (+)	99%	Field-mosquito	Panama Metro
Betaproteobacteria	*Chromobacterium sp.*	*Chromobacterium haemolyticum*	Gram (−)	98%	Field-mosquito	Chorrera
Betaproteobacteria	*Comamonas sp.*	*Comamonas testosteroni*	Gram (−)	97%	Field-mosquito	Panama Metro
Betaproteobacteria	*Comamonas sp.*	*Comamonas testosteroni*	Gram (−)	99%	Field-mosquito	Panama Metro
Betaproteobacteria	*Shinella*	*Shinella kummerowiae*	Gram (−)	98%	Field-mosquito	Panama Metro
Flavobacteria	*Elizabethkingia sp.*	*Elizabethkingia meningoseptica*	Gram (−)	99%	Lab-mosquito	Maryland, USA
Flavobacteria	*Chryseobacterium sp.*	*Chryseobacterium sp. ISE14*	Gram (−)	98%	Field-mosquito	Panama Metro
Gammaproteobacteria	*Acinetobacter sp.*	*Acinetobacter sp.18N3*	Gram (−)	99%	Field-mosquito	Chorrera
Gammaproteobacteria	*Aeromonas sp.*	*Aeromonas hydrophila strain S1*	Gram (−)	99%	Field-mosquito	Chorrera
Gammaproteobacteria	*Aeromonas sp.*	*Aeromonas sp. WC56*	Gram (−)	199%	Field-mosquito	Chorrera
Gammaproteobacteria	*Enterobacter sp.*	*Enterobacter hormaechei*	Gram (−)	99%	Field-mosquito	Chorrera
Gammaproteobacteria	*Enterobacter sp.*	*Enterobacter hormaechei subsp. Steigerwaltii*	Gram (−)	99%	Field-mosquito	Panama Metro
Gammaproteobacteria	*Enterobacter sp.*	*Enterobacter ludwigii strain GTR*	Gram (−)	99%	Field-mosquito	Panama Metro
Gammaproteobacteria	*Shigella sp.*	*Shigella sp. SZ012*	Gram (−)	99%	Field-mosquito	David
Gammaproteobacteria	*Pantoea sp.*	*Pantoea dispersa ND4*	Gram (−)	99%	Field-mosquito	Panama Metro
Gammaproteobacteria	*Pantoea sp.*	*Pantoea agglomerans strain AR_PINLBH4*	Gram (−)	99%	Lab-mosquito	Maryland, USA
Gammaproteobacteria	*Pantoea sp.*	*Pantoea dispersa 5BJN1*	Gram (−)	99%	Field-mosquito	Panama Metro
Gammaproteobacteria	*Proteus sp.*	*Proteus mirabilis*	Gram (−)	100%	Field-mosquito	Chorrera, Panama Metro
Gammaproteobacteria	*Proteus sp.*	*Proteus penneri*	Gram (−)	99%	Field-mosquito	Chorrera
Gammaproteobacteria	*Pseudomonas sp.*	*Pseudomonas sp. M2L4*	Gram (−)	99%	Field-mosquito	Chorrera
Gammaproteobacteria	*Pseudomonas sp.*	*Pseudomonas stutzeri strain 1-1*	Gram (−)	98%	Field-mosquito	Panama Metro
Gammaproteobacteria	*Serratia sp.*	*Serratia marcescens strain N1.14*	Gram (−)	99%	Field-mosquito	Panama Metro
Gammaproteobacteria	*Serratia sp.*	*Serratia marcescens strain P3*	Gram (−)	99%	Lab-mosquito	Maryland, USA
Gammaproteobacteria	*Leclercia sp.*	*Leclercia sp. 1185/07*	Gram (−)	99%	Field-mosquito	David

16S ribosomal RNA (rRNA) gene sequences was used to study phylogenetic affiliations of midgut bacteria.

### Certain field mosquito midgut-associated bacterial species significantly impair dengue virus infection

**Figure 3:**
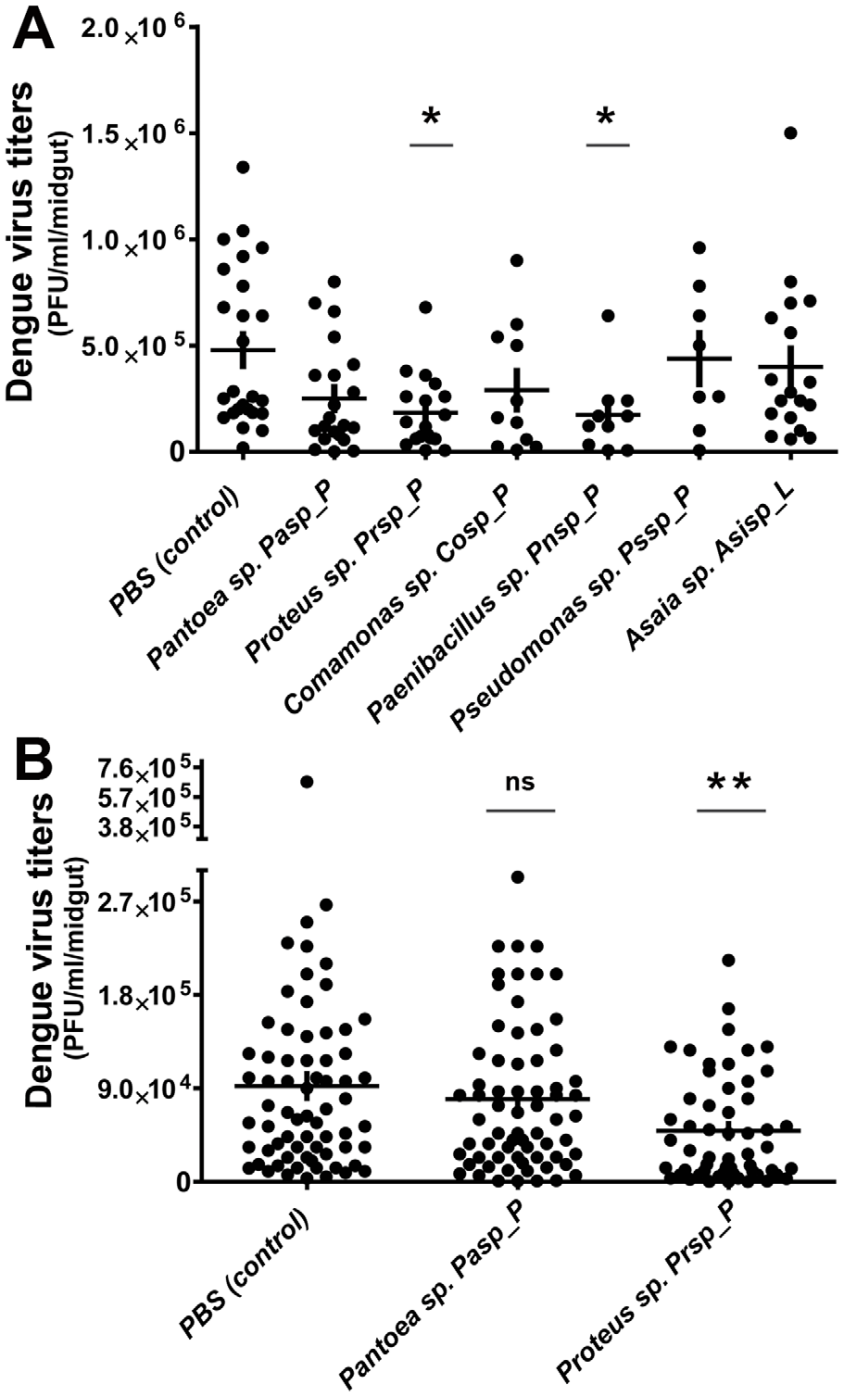
Bacterial influence of dengue virus infection in the mosquito midgut. Dengue virus (DENV-2) loads in mosquito midguts after the introduction of a single-bacterium isolate (through blood meal (**A**) and sugar meal (**B**) into the mosquito midgut, as compared with control mosquitoes (PBS). Data were analyzed using a one-way ANOVA, followed by Dunnett's post-test; *, p<0.05; **, p<0.001.

To investigate whether certain bacteria isolated from field mosquitoes might influence dengue virus infection of the midgut, we conducted bacterial reintroduction assays through a blood meal or sugar meal (Figure 2B and Figure 2C) prior to dengue virus infection. Recolonization of mosquito midguts, previously rendered aseptic through antibiotic treatment, with single-isolate bacteria through a blood meal led to a marked decrease in viral titers in the midgut at 7 days post-bloodmeal (PBM). Introduction of two bacteria species (*Proteus sp.* Prpsp_P *and Paenibacillus sp* Pnsp_P) separately into the mosquito midguts resulted in a significantly lower level of dengue virus infection, while introduction of other species (among them *Pantoea sp.* Pasp_P and *Comamonas sp.* Cosp_P) produced no significant difference in dengue virus titer from that of control group mosquitoes (Figure 3A).

Next we wanted to assess the impact of selected bacteria on dengue virus infection when introduced through a nectar meal, since this would be the most likely route of introduction in the field and exposure in a potential future symbiotic biocontrol strategy. The current perception is that the ingested nectar meal is stored in the mosquito crop and then relocated to the midgut for digestion [19], [20]. To determine the location of the ingested sugar meal in the mosquito's digestive system, we exposed mosquitoes to a food color-dyed sugar meal. Following a 6-h exposure to the dyed-sugar meal, the blue sugar meal could be observed in the crop and midgut of some mosquitoes, while the remaining mosquitoes showed the presence of the sugar meal only in the midgut (Figure S2). At the end of a 24-h exposure, all mosquitoes were found to have food color-dyed sugar meal in both the midgut and crop.

To assess the successful colonization of the mosquito midgut by the reintroduced bacteria, mosquito midguts were dissected, homogenized, and plated on LB agar at 3 days post-bacterial acquisition and prior to the time point at which dengue virus infection normally occurs. We observed a high prevalence of *Proteus sp.* Prsp_P (100%) and a somewhat lower prevalence (69%) of *Pantoea sp*. Pasp_P in the midgut of the mosquitoes (Figure 2B and Figure 2C).

Reintroduction of *Proteus sp* Prsp_P into the midgut through a sugar meal led to a significant decrease in dengue virus titers, but no significant effect on dengue virus infection was observed in mosquitoes colonized by *Pantoea sp.* Pasp_P (Figures 3B).

### Midgut bacteria from field-derived mosquitoes induces local and systemic immune gene expession

**Figure 4:**
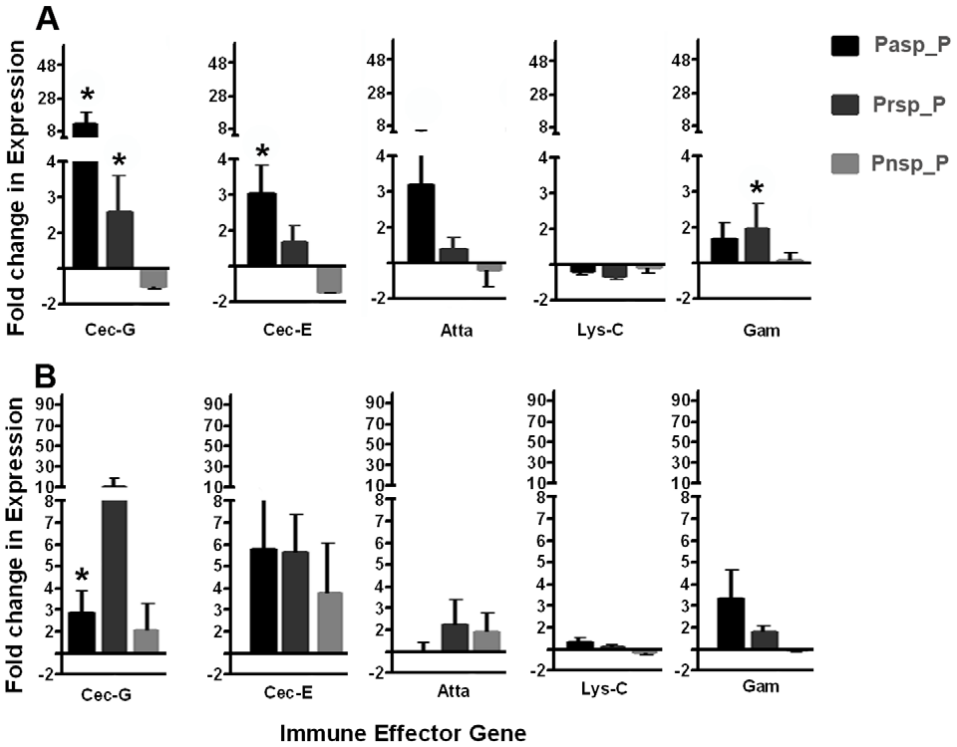
Antimicrobial peptide gene transcript abundance upon midgut exposure to selected bacterial isolates. Fold change in the transcript abundance of selected antimicrobial peptide genes in the midgut (**A**) and fat body (**B**) of mosquitoes 2 days after the introduction, via a sugar meal, of either *Pantoea sp.* Pasp_P, *Proteus sp.* Prsp_P, or *Paenibacillus sp.* Pnsp_P. Data was analyzed by one-way ANOVA with Dunnett's post-test; *, p<0.05.

Reintroduction of isolated bacteria into the antibiotic-treated (aseptic) mosquitoes' midguts elicited changes in transcript abundace of a number of antimicrobial peptide genes, including cecropin, gambicin and attacin in the midgut (Figure 4A) and the abdominal fat body tissue (Figure 4B). This result suggests that modulation of immune gene transcript abundance by the reintroduced bacteria could have a detrimental effect on dengue virus infection.

### Effects of dengue virus infection on the mosquito midgut microbiota and antimicrobial peptide geen expression

**Figure 5:**
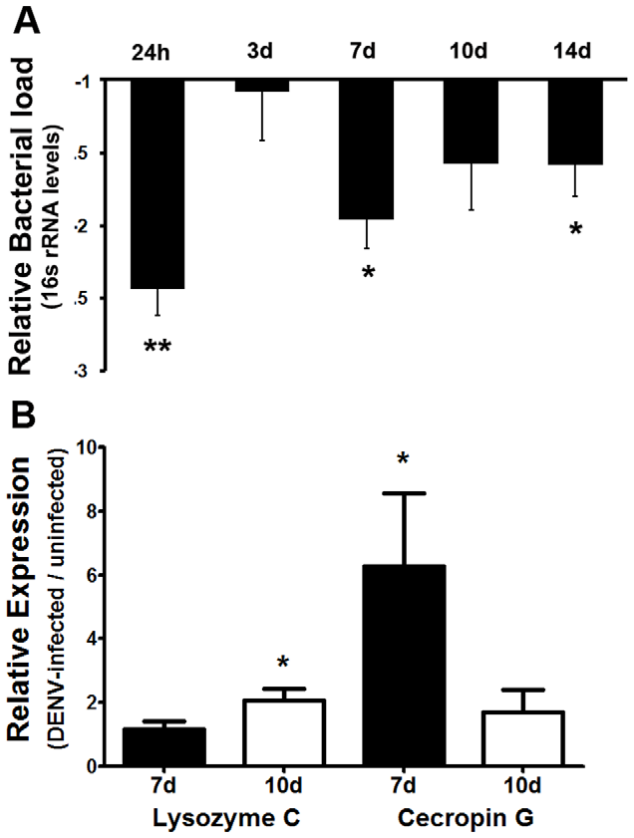
Dengue virus infection modultaes the mosquito midgut microbiota. (**A**) Total bacterial 16s RNA levels in the midguts of dengue virus-infected mosquitoes relative to those of uninfected mosquitoes. Bacterial loads were assessed by qPCR from pools of 10 midguts per replicate, and at least 4 independent biological replicates were included. Data were analyzed by one-way ANOVA with Dunnett's post-test; *, p<0.05. (**B**) Antimicrobial peptide gene transcript abundance in the midgut of dengue virus-infected mosquitoes relative to uninfected mosquitoes at 7 days and 10 days post-infection. Data were analyzed by Mann-Whitney U-test; *, p<0.05.

Dengue virus infection of the mosquito's midgut led to significant decrease in the overall bacterial load (as assessed by 16s rRNA transcript levels) at 24 h, 7 days, and 14 days after ingestion of a dengue virus-supplemented blood meal. Interestingly, the difference in the bacterial 16s rRNA transcript levels between dengue virus-infected and uninfected mosquitoes was less prominent at 3 days post-infection (Figure 5A). Analysis of the relative transcript abundance of the antimicrobial peptide genes lysozyme C, and cecropin G revealed that cecropin G transcripts were significantly elevated in dengue-infected mosquitoes at 7 days post-infection but showed no difference from control levels at 10 days post-infection. Lysozyme C also showed a transient changes in transcript abundace, with no difference from control levels at 7 days but significant changes at 10 days post-infection (Figure 5B).

### Dengue infection responsive antimicrobial peptide genes influences the mosquito midgut bacterial load

**Figure 6:**
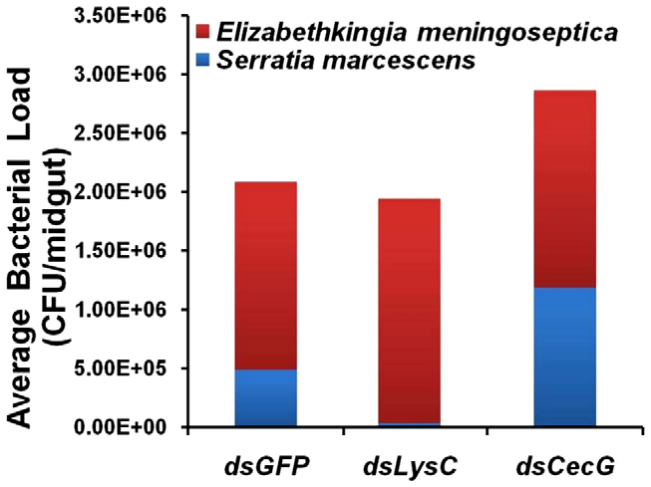
Effect of antimicrobial peptide gene silencing on themidgut microbiota bacterial species composition. Bacterial composition in the midguts of lysozyme C, cecropin and GFP silenced mosquitoes at 3 days post-dsRNA injection. Two main bacterial types were observed in each group of mosquitoes. Data represent the microbial composition of 2 independent biological replicates (n = 20).

**Figure 7:**
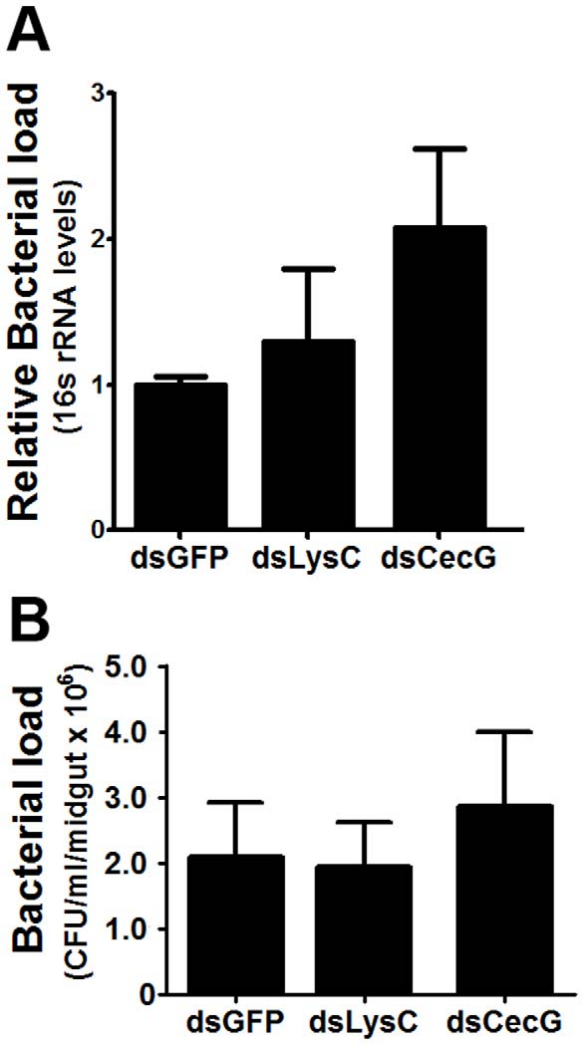
Bacterial loads in mosquito midguts following antimicrobial peptide gene silencing. Bacterial load was assessed by a (**A**) culture-independent method and (**B**) culture-dependent method. Data were analyzed by one-way ANOVA with Dunnett's post-test; *, p<0.05.

To assess the involvement of antimicrobial effector genes in regulating the midgut microbiota, we employed an RNAi-based gene silencing approach in conjunction with CFU assays. Although not statistically significant,silencing of several effector genes led to changes in the growth of the midgut bacterial populations compared to the control group (GFP dsRNA-injected mosquitoes) (Figure 6). This suggests that one function of these immune factors is to maintain a basal level of immunity to control microbial proliferation. Interestingly, we did not observe a significant increase in the midgut bacterial load after silencing the cecropin G and lysozyme C genes, suggesting that these factors may play more specialized roles in immunity (Figure 7A and 7B).

### Antimicrobial peptide genes influence mosquitoes' susceptibility to dengue virus infection of the midgut

**Figure 8:**
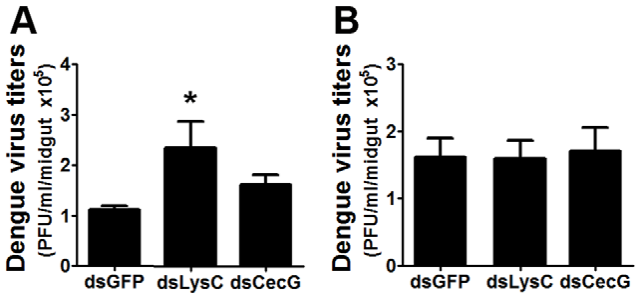
Dengue virus titers following antimicrobial peptide gene silencing. Dengue viral loads were assessed from (**A**) septic and (**B**) aseptic (antibiotic-treated) mosquito midguts. Data were analyzed by one-way ANOVA with Dunnett's post-test; *, p<0.05.

We used a RNAi-based gene silencing approach to assess the effect of selected antimicrobial peptide genes on dengue virus infection, some of which are known to be regulated by our field-derived bacteria. This treatment led to an overall increase in dengue virus titers in the mosquito midgut especially for lysozyme C, suggesting that this gene might exert a significant inhibitory effect, on dengue virus infectivity (Figure 8A). However, this effect was lost when the mosquitoes were maintained aseptically with antibiotics prior to receiving an infectious blood meal (Figure 8B). This might indicate that the infection phenotype observed upon lysozyme C–silencing reflects an indirect effect. It is possible that lysozyme inhibit the growth of bacteria that are beneficial to the virus, or, alternatively lysozyme may act against bacteria that compete with other bacteria that have a detrimental effect on the virus. The current analysis does not allow for a detailed mechanistic insight on this.

## Discussion

During their life span, insects harbor a variety of microbes in their intestine, some of which are needed for successful growth to adulthood, and some as aids in digestion, nutrition, and reproduction [21] as well as protection against pathogens [10], [22]–[25]. This situation is especially true for mosquitoes that, as larvae, develop in stagnant microbe-rich water, feeding on various bacteria and fungi, and that as adults are exposed to microbes, parasites, and viruses through plant nectars and ingested blood. For example, it has been shown that antibiotic treated aseptic *Anopheles gambiae* mosquitoes are more susceptible to *Plasmodium* infection and possess a lower basal level of immune gene transcripts than do *An. gambiae* with a normal microbial population [26], [27]. The basal level of immune activity appears to be critical in defining the level of susceptibility to *Plasmodium* infection [28].

With regard to virus-mosquito interactions, the intracellular bacterium *Wolbachia spp*. has been shown in several studies to affect dengue virus infection in *Ae. aegypti* mosquitoes [29] and infection with the Japanese encephalitis virus in aseptic *Culex bitaeniorhyncus* [30]. We have previously shown that mosquitoes with a reduced midgut bacterial load (as a result of antibiotic treatment) can support higher dengue infection levels than can septic mosquitoes [11]. Furthermore, the antibiotic-treated aseptic mosquitoes display a lower basal level of several Toll pathway-related genes transcripts. We have shown that the Toll pathway is involved in the anti-dengue defense [11]. We cannot, however, exclude other possible mechanisms by which the bacteria may hinder virus infection in the mosquito.

In order to assess this phenomenon in greater detail and select bacteria that can mediate potent anti-dengue activity and meet other criteria (easily cultivable and major representation in the midgut microbiome) for the development of dengue biocontrol strategies, we have now isolated and characterized cultivable bacteria from the midguts of field mosquitoes collected in dengue-endemic areas of Panama. Bacterial isolates from field collections belonged to several phylogenetic classes, but no predominant genus was observed. Many of these bacterial species have been previously isolated from mosquitoes and may be better adapted to the mosquito midgut environment. The diversity of microbes isolated from field mosquitoes suggests a complex mosquito midgut microbiome that is likely to affect the outcome of infection and the mosquito's midgut immune homeostasis. Our midgut bacteria discovery method identified only live, replicating bacteria that could grow aerobically on a rich culture medium, and this approach likely explains some of the discrepancies between our results and those of studies that have employed PCR-based amplification of bacterial DNA, much of which may have been derived from dead, minor, and/or transient microbial constituents of the midgut microflora [15], [31].

Reintroduction of some of these bacterial species through a blood meal led to changes in susceptibility of the midgut tissue to dengue infection. Furthermore, reintroduction of bacterial isolates via a sugar meal into the midgut of *Ae. aegypti* mosquitoes resulted in a significant decrease in dengue virus infection in the case of one bacterial isolate, *Proteus sp.* Prsp_P.

These bacteria may either indirectly exert an anti-dengue effect by boosting basal immunity or may directly influence the virus' infectivity. The bacteria could, for example, act prior to dengue virus infection of the midgut via bacterial metabolites that are detrimental to the dengue virus, or act as a barrier for the virus via steric hindrance, by growing along the midgut epithelium [15].

In contrast to the effects produced by *Proteus sp*. Prsp_P, reintroduction of *Pantoea sp.* Pasp_P had no effect on dengue virus infection, perhaps because of the inability of this bacterium to effectively colonize the mosquito's midgut.

This could partially offset the anti-dengue effects that derive from the elicitation of the mosquito's immune system by this bacterium. Alternatively, although Pasp_P shows a slightly higher immune induction than Prsp_P, our gene expression assays only addressed one time point of amp transcript abundance, and it is quite likely that Prsp_P may elicit an overall stronger induction of these genes over an extended time period. It is also possible that Prsp_P induces some other unknown anti-viral factor stronger than Pasp_P.

Furthermore, given that our introduction of bacteria was performed with a single bacterial species at a time, it is possible that lack of effect on dengue virus infection was because this bacterium needs to act in synergy with other microbes of the midgut. This type of synergistic effects may also alter some of our observed ant-dengue activities for the other studied bacteria, when combined with multiple bacterial species.

Our analyses of immune gene expression in mosquitoes exposed to the studied bacteria revealed responses that were similar in their direction of regulation but different in their magnitude. We observed elevated immune gene transcripts in both the midgut and fat body tissues, thus pointing to a local as well as a systemic immune response. These two compartment-specific responses could act in concert to limit dengue virus infection and dissemination in the mosquito host. The transcript abundance of the antimicrobial peptides we assayed has been shown to be regulated by the immune signaling pathways that govern the defense against dengue virus infection [11], [32], [33]. Thus, it is possible that mosquito immune responseselicited by the bacteria play a significant role in reducing the level of dengue infection in the mosquito midgut. In fact, recently, a cecropin-like peptide with anti-dengue virus properties was found to be elicited in the salivary gland of dengue virus-infected mosquitoes [34] and cecropin-D and defensin-C peptides have been shown to have anti-dengue activity in the mosquito midgut [35].

The mosquito can be considered a holobiont unit, in which the mosquito, its midgut microflora, and the dengue virus are involved in complex reciprocal tripartite interactions. Our analysis of these interactions has indicated that dengue infection in the mosquito is able to elicit an immune response involving the elevated transcript abundance of antimicrobial peptide genes such as cecropin, attacin, and lysozyme C [11], [32], [33]. Even though the antiviral activity of the mosquito's antimicrobial peptides have yet to be characterized, a cecropin-like peptide was recently found to have anti-dengue virus activity [34]. In addition, antimicrobial peptides are effective in controlling bacteria [36]–[38], and their elicitation by dengue virus infection can therefore modulate the mosquito's midgut microflora. Our observations agree with this assertion, in that dengue virus-infected mosquito midguts displayed a lower bacterial load (as measured by 16s rRNA) than did those of uninfected mosquitoes.

In summary, our analysis of the reciprocal interactions between the dengue virus, mosquito immune system, and bacteria isolated from midguts of field mosquitoes collected in Panama has revealed a marked decrease in viral load in mosquitoes infected with certain natural bacterial isolates. Transcript abundance analysis of selected antimicrobial peptide genes suggested that the mosquito's microbiota elicits an immune response that appears to act in part to control dengue infection. In turn, the activation of the immune system by dengue virus infection potentiates the mosquito's immune homeostasis and suppresses the microbiota of its midgut. A better understanding of these complex reciprocal interactions may facilitate the development of novel biocontrol strategies for dengue transmission.

## Supporting Information

Figure S1 **Representative panel of bacterial isolates identified by colony morphology and color.** (A) *Leclercia sp.* Lesp_P; (B) *Pseudomonas sp.* Pssp_P; (C) *Pantoea sp.* Pasp_P (yellowish) and *Serratia sp.* (white); (D) Asaia sp. Asisp_L; (E) Proteus sp. Prsp_P; (F) Micrococcus sp. Mcsp_P; (G) *Pseudomonas sp.* Ps2sp_P (yellow) and *Paenibacillus sp*. Pnsp_P(white) and (H) *Chromobacterium sp.* Csp_P.
(TIF)

Figure S2 **Introduction of a sugar meal into the mosquito midgut and crop.** Comparison of mosquito midgut and crop at 6 h after exposure to a food color-dyed sugar meal.
(TIF)

Table S1 **PCR primers used in gene expression analyses.**
(PDF)

Table S2 **PCR primers used to amplify gene segments for the production of dsRNA segments.**
(PDF)
