# Supplementary figures and images for: Reciprocal Tripartite Interactions between the Aedes aegypti Midgut Microbiota, Innate Immune System and Dengue Virus Influences Vector Competence
Source: PLoS Negl Trop Dis. 2012 Mar 6;6(3):e1561. doi: 10.1371/journal.pntd.0001561 (PMC3295821; doi:10.1371/journal.pntd.0001561)

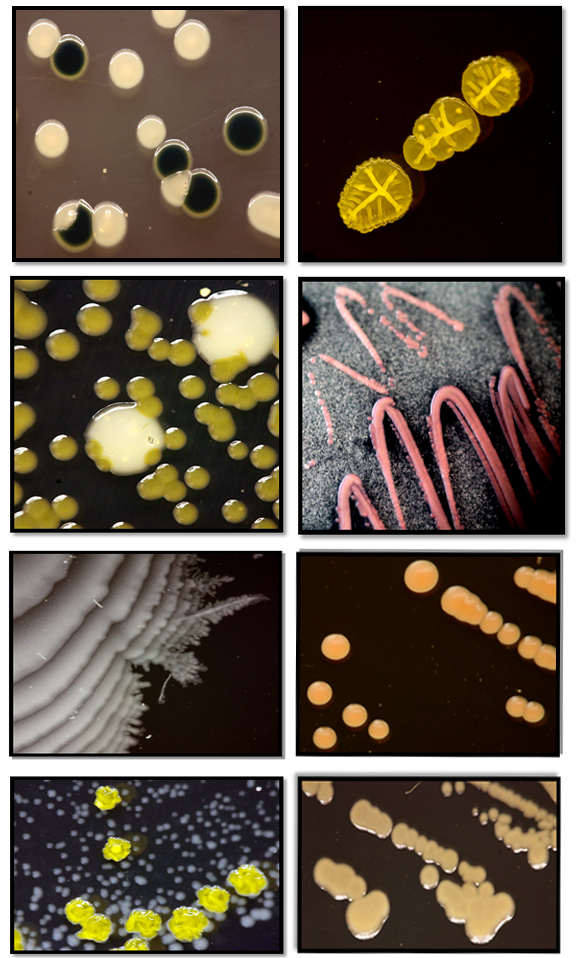

Supplement: Figure S1 — Representative panel of bacterial isolates identified by colony morphology and color. (A) Leclercia sp. Lesp_P; (B) Pseudomonas sp. Pssp_P; (C) Pantoea sp. Pasp_P (yellowish) and Serratia sp. (white); (D) Asaia sp. Asisp_L; (E) Proteus sp. Prsp_P; (F) Micrococcus sp. Mcsp_P; (G) Pseudomonas sp. Ps2sp_P (yellow) and Paenibacillus sp. Pnsp_P(white) and (H) Chromobacterium sp. Csp_P. (TIF) [file pntd.0001561.s001.tif]

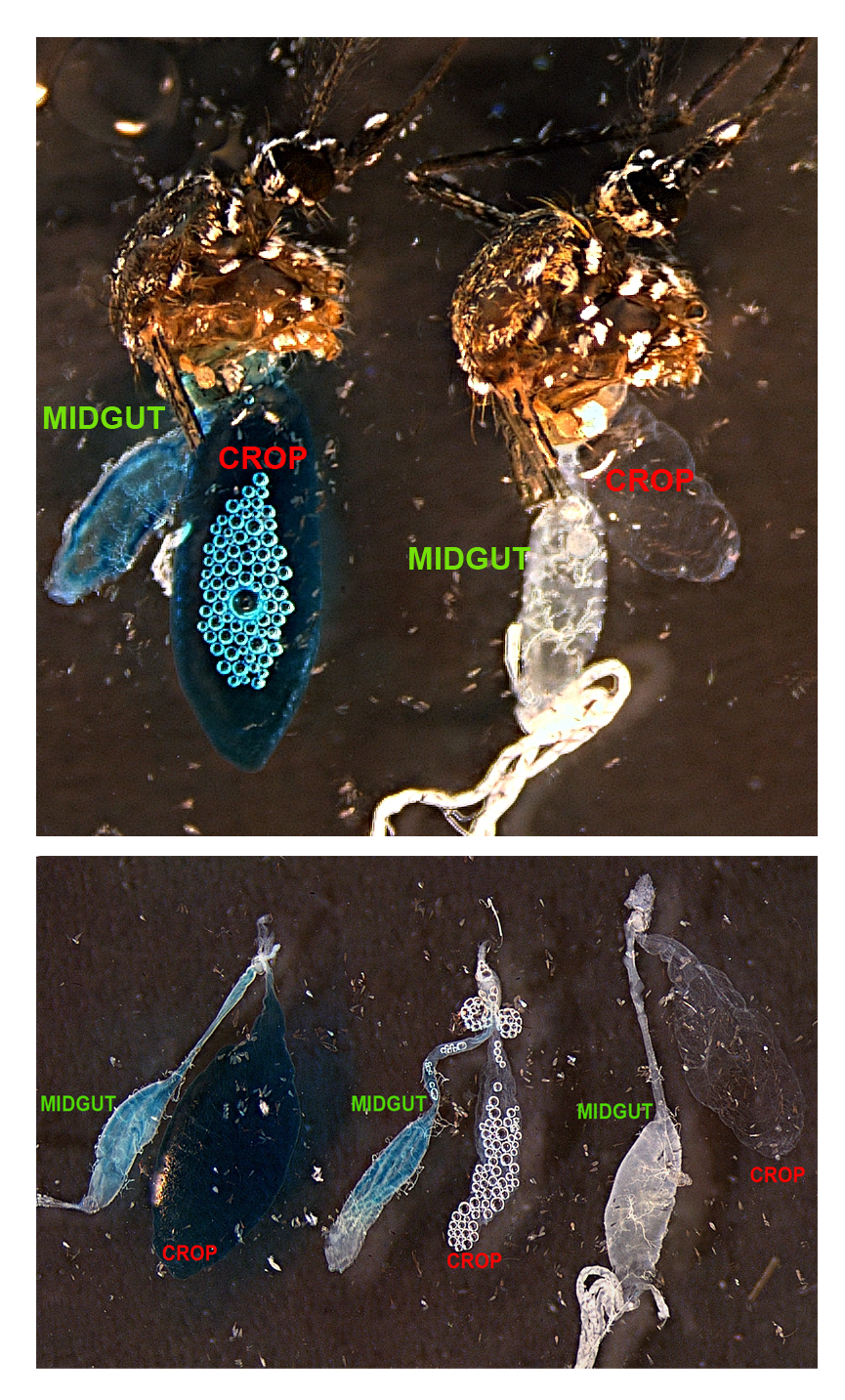

Supplement: Figure S2 — Introduction of a sugar meal into the mosquito midgut and crop. Comparison of mosquito midgut and crop at 6 h after exposure to a food color-dyed sugar meal. (TIF) [file pntd.0001561.s002.tif]
