# Supplementary material for: Reciprocal Tripartite Interactions between the Aedes aegypti Midgut Microbiota, Innate Immune System and Dengue Virus Influences Vector Competence
Source: PLoS Negl Trop Dis. 2012 Mar 6;6(3):e1561. doi: 10.1371/journal.pntd.0001561 (PMC3295821; doi:10.1371/journal.pntd.0001561)
Supplement: Table S1 — PCR primers used in gene expression analyses. (PDF) [file pntd.0001561.s003.pdf]

Gene/Transcript ID	Primer name	Sequence
AAEL003389	Attacin Forward	5'-TTGGCAGGCACGGAATGTCTTG-3'
	Attacin Reverse	5'-TGTTGTCGGGACCGGGAAGTG-3'
AAEL009496	Ribosomal S7 Forward	5'-GGGACAAATCGGCCAGGCTATC-3'
	Ribosomal S7 Reverse	5'-TCGTGGACGCTTCTGCTTGTTG-3'
AAEL004522	Gambicin Forward	5'-GCCAAAACCTGTTCCTCTTG-3'
	Gambicin Reverse	5'-CGATGTAGCATTCGGTGATG-3'
AAEL015515	Cecropin-G Forward	5'-CCAAGCCTTGTGAACCAGTA-3'
	Cecropin-G Reverse	5'-GGCCACCTGCTTCAGACT-3'
AAEL000611	Cecropin-E Forward	5'-CGAAGCCGGTGGTCTGAAG-3'
	Cecropin-E Reverse	5'-ACTACGGGAAGTGCTTTCTCA-3'
AAEL015404	Lysozyme C Forward	5'-CCACGGCAACTGGATATGTCT-3'
	Lysozyme C Reverse	5'-TCTGCGTCACCTTGGTGGTAT-3'
