# Supplementary material for: Reciprocal Tripartite Interactions between the Aedes aegypti Midgut Microbiota, Innate Immune System and Dengue Virus Influences Vector Competence
Source: PLoS Negl Trop Dis. 2012 Mar 6;6(3):e1561. doi: 10.1371/journal.pntd.0001561 (PMC3295821; doi:10.1371/journal.pntd.0001561)
Supplement: Table S2 — PCR primers used to amplify gene segments for the production of dsRNA segments. (PDF) [file pntd.0001561.s004.pdf]

Gene/Transcript ID	Primer name	Sequence
AAEL015404	dsLysC-F	TAATACGACTCACTATAGGGACATTCAGCGAAGCCAAAGT
	dsLysC-R	TAATACGACTCACTATAGGGAAATGTTGGGCAGTTCCTTG
AAEL015515	dsCecG-F	TAATACGACTCACTATAGGGTCCAAGCCTTGTGAACCAGT
	dsCecG-R	TAATACGACTCACTATAGGGTTAGCCCCAGCTACAACAGG
